# Engineered DNA-encoded monoclonal antibodies targeting *Plasmodium falciparum* circumsporozoite protein confer single dose protection in a murine malaria challenge model

**DOI:** 10.1038/s41598-022-18375-6

**Published:** 2022-08-22

**Authors:** Nicholas J. Tursi, Sophia M. Reeder, Yevel Flores-Garcia, Mamadou A. Bah, Shamika Mathis-Torres, Berenice Salgado-Jimenez, Rianne Esquivel, Ziyang Xu, Jacqueline D. Chu, Laurent Humeau, Ami Patel, Fidel Zavala, David B. Weiner

**Affiliations:** 1 Vaccine and Immunotherapy Center, The Wistar Institute, Philadelphia, PA 19104 USA; 2 Perelman School of Medicine, University of Pennsylvania, Philadelphia, PA 19104 USA; 3 Department of Molecular Microbiology and Immunology, Malaria Research Institute, Bloomberg School of Public Health, Johns Hopkins University, Baltimore, MD 21205 USA; 4 Inovio Pharmaceuticals, Plymouth Meeting, PA 19462 USA

**Keywords:** DNA vaccines, Gene delivery

## Abstract

Novel approaches for malaria prophylaxis remain important. Synthetic DNA-encoded monoclonal antibodies (DMAbs) are a promising approach to generate rapid, direct in vivo host-generated mAbs with potential benefits in production simplicity and distribution coupled with genetic engineering. Here, we explore this approach in a malaria challenge model. We engineered germline-reverted DMAbs based on human mAb clones CIS43, 317, and L9 which target a junctional epitope, major repeat, and minor repeat of the *Plasmodium falciparum* circumsporozoite protein (CSP) respectively. DMAb variants were encoded into a plasmid vector backbone and their expression and binding profiles were characterized. We demonstrate long-term serological expression of DMAb constructs resulting in in vivo efficacy of CIS43 GL and 317 GL in a rigorous mosquito bite mouse challenge model. Additionally, we engineered an Fc modified variant of CIS43 and L9-based DMAbs to ablate binding to C1q to test the impact of complement-dependent Fc function on challenge outcomes. Complement knockout variant DMAbs demonstrated similar protection to that of WT Fc DMAbs supporting the notion that direct binding to the parasite is sufficient for the protection observed. Further investigation of DMAbs for malaria prophylaxis appears of importance.

## Introduction

According to the World Health Organization, there were approximately 241 million malaria cases globally, resulting in 627,000 deaths, in 2020^1^. The majority of malaria cases occur in Africa, Southeast Asia, and South America^2^. Multiple species of the *Plasmodium* parasite, the causative agent of malaria, infect humans which result in patient symptoms ranging from fever and chills to anemia, coma, and death^3^. In Africa, *Plasmodium falciparum* accounts for the majority of malaria cases.

There have been numerous attempts to develop malaria vaccines or therapeutics targeting different aspects of the parasite life cycle^4^. The pre-erythrocytic stage occurs after parasite injection by mosquitoes and before liver invasion. The predominant surface protein of the sporozoite is the circumsporozoite protein (CSP). CSP is associated with infectivity of sporozoites and contains both B and T cell epitopes implicated in protection^5–8^. RTS,S/AS01, a new malaria vaccine, is now approved by the World Health Organization and utilized under the trade name Mosquirix. Phase III trial results demonstrated that vaccination with RTS,S, which contains portions of the central repeat domain and C terminal T cell epitopes of *Plasmodium falciparum* CSP (PfCSP) scaffolded to the Hepatitis B virus surface antigen (HBsAg), induced a reduction in malaria cases in children over a span of 5 years^4,9–11^. A newer generation vaccine, R21, contains one quarter of the HBsAg as the original RTS,S, and has demonstrated upwards of 77% efficacy in a trial in Burkina Faso when formulated in Matrix M^12,13^. Another approach is a vaccine developed by Sanaria, PfSPZ, which consists of attenuated whole sporozoites. One distinct advantage of PfSPZ is that it presents PfCSP in its native conformation; PfSPZ has demonstrated partial protection in a Phase 1 trial but no significant protection in a Phase 2 trial in children^14–16^. Additionally, the use of PfSPZ with the anti-malarial drug chloroquine has demonstrated heterologous protection in malaria naïve individuals in a recent trial^17^. Correlates of protection from irradiated sporozoites include both antibody and T cell responses^18,19^. However, challenges with sporozoite vaccine models persist, as sporozoites may not be effective at priming immune responses if over-irradiated or an active infection may ensue if under-irradiated^20^, supporting the notion that additional vaccines or prophylaxis approaches are likely important.

Monoclonal antibodies (mAbs) have also been isolated from malaria vaccinees and have defined sites of vulnerability on the CSP antigen. These include the human mAbs CIS43, 317, and L9^21–23^. CIS43 is a human IgG1 mAb isolated from a volunteer immunized with the PfSPZ vaccine. This mAb exhibits preferential binding to a distinct epitope spanning the N terminus and NANP repeat junction of PfCSP as well as binding to the classically immunodominant NANP repeat region. Both passive transfer and adeno-associated virus delivery of CIS43 demonstrated protection in murine challenge models of malaria^21,24^. Importantly, a half-life extended variant of CIS43, CIS43LS, has demonstrated preliminary efficacy in a controlled human malaria challenge in a Phase I clinical trial^25^. 317 is a major repeat (NPNA) targeting human mAb that was isolated from a controlled human malaria infection trial of RTS,S immunized individuals. 317 has demonstrated potent protection in murine challenge models and along with a sister clone 311 these antibodies have elucidated important structural information about the NANP repeat region through Fab binding^22^. Additionally, L9 is a minor (NVDP) repeat targeting mAb that was isolated using a probe targeting the junctional region of CSP from PfSPZ-immunized individuals. L9 has demonstrated protection in mouse models through sporozoite neutralization in the liver^23^. As recombinant mAbs, CIS43, 317, and L9 have all demonstrated promising results in preclinical and, in the case of CIS43, CHMI models.

Recombinant mAbs are becoming more common for infectious disease prophylaxis or therapy. TY014 is a human therapeutic mAb targeting yellow fever currently being evaluated in Phase I clinical trials^26^ (ClinicalTrials.gov NCT03776786). Additionally, numerous anti-SARS-CoV-2 monoclonal antibody cocktails have been granted Emergency Use Authorization to treat or prevent COVID-19^27–29^. However, there are distinct technological limitations for global human deployment, including the high cost of production and the short half-life of such biologics in vivo^30,31^. Genetic approaches such as DNA-encoded monoclonal antibodies (DMAbs) are directly launched in vivo and result in systemic, prolonged circulation of functional antibodies^30^. DMAb administration results in several months of in vivo mAb expression, a potential advantage for endemic and cyclic infections. DMAbs have been recently demonstrated promise for specific viral and bacterial pathogens^32–37^, and may be of benefit against parasitic infections like malaria.

We previously described the use of nucleotide and sequence engineering to improve in vivo DMAb expression^33,34,38^. Additionally, we have also shown that DMAbs can be Fc modified to enhance or ablate complement activity in a *N. gonorrhoeae* model with improved clearance in bacterial challenge^39^. Modification of antibody Fc domains through two alanine substitutions ablates IgG1 hexamerization and subsequent affinity for C1q and complement deposition^40^. In terms of malaria prophylaxis, reports have demonstrated that complement fixing anti-CSP antibody titers in children correlates with protection and wanes quickly over time^41,42^. Additionally, sporozoite immunization-induced IgG antibodies had enhanced activity against heterologous sporozoites with complement present in vitro^43^. Yet, the effect of complement abrogating mutations on protection has not been determined in the context of anti-sporozoite stage mAbs. As DMAbs are capable of being rapidly sequence modified for assessing in vivo efficacy, we sought to use the DMAb approach to test whether complement engagement is necessary for the protective efficacy conferred by sporozoite stage mAbs.

Here, we describe the design and development of anti-PfCSP DMAbs based on recombinant pre-erythrocytic mAbs CIS43, 317, and L9 and their evaluation in vitro, in vivo, and in malaria challenge models. Following a proof-of-concept study developing germline-modified antibody variant based on CIS43, CIS43 GL, which resulted in potent in vivo expression, we next advanced these into malaria challenge. We observed that the DMAbs demonstrated similar inhibition of liver infection as the positive control monoclonal antibody, mAb 311^22^ in an acute mosquito bite challenge model. DNA-encoded germline modified variants of mAbs 317 and L9, 317 GL and L9 GL respectively, were also generated and demonstrated long term serological expression in vivo*.* In this analysis, both CIS43 GL and 317 GL demonstrated significant protection in an extended mosquito bite challenge model. Finally, modifications to CIS43 GL and L9 GL were engineered to ablate C1q affinity to test the importance of Fc function and complement deposition to the protection elicited by anti-malarial DMAbs. Complement knockout variants demonstrated similar protection in mosquito bite challenge compared to wild-type controls supporting the notion that the mechanism of protection is direct binding of the antibody to relevant CSP epitopes in this challenge model.

## Results

### Characterization of germline-modified variant CIS43 GL DMAb

**Figure 1:**
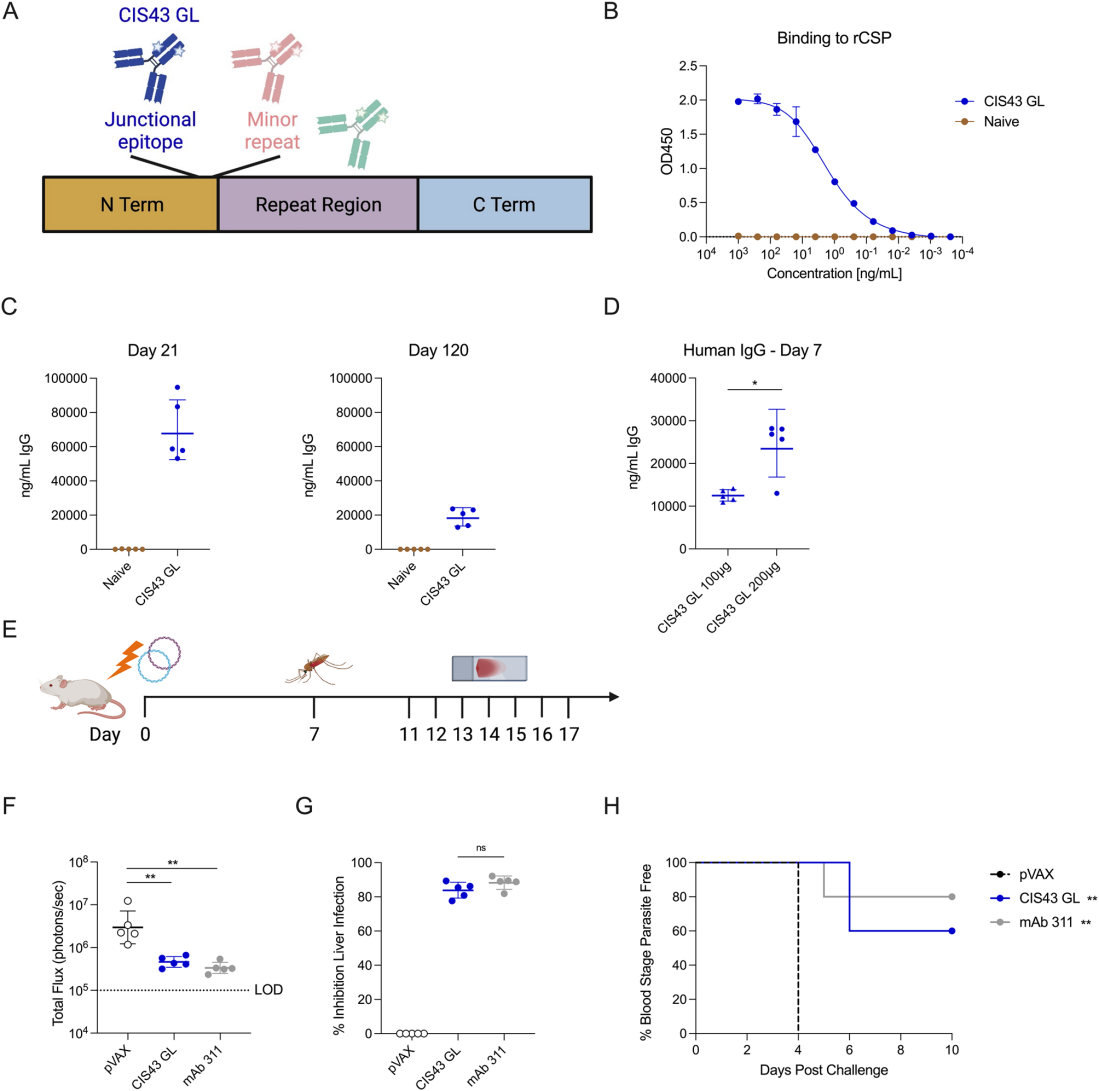
Proof-of-concept CIS43 GL DMAb protects mice in mosquito bite challenge. (**A**) Schematic demonstrating CIS43 GL binding domain on CSP. (**B**) Binding ELISA of pooled supernatants harvested from Expi293F transfection to rCSP (mean ± SD). (**C**) In vivo expression of CIS43 GL in BALB/c mice at the peak expression timepoint Day 21 (left) through Day 120 (right). Mice were immunized with 100 µg CIS43 intramuscularly in the tibialis anterior with CD4+/CD8+ T cell depletion (n = 5 mice/group; geometric mean ± geometric SD). (**D**) In vivo expression of CIS43 GL in BALB/c mice without CD4+/CD8+ T cell depletion (n = 5 mice/group; geometric mean ± geometric SD). Mice were administered 100 µg or 200 µg intramuscularly in the tibialis anterior and/or quadricep. *p = 0.032 by two-tailed Mann–Whitney test. (**E**) Experimental layout of challenge. Mice were immunized with CIS43 GL and challenged 7 days post administration with mosquitos carrying *PbPfLuc* parasites. Positive control recombinant mAb 311 was administered 16 h prior to challenge. Blood smears for blood stage parasitemia were performed from days 4–10 post challenge. (**F**) Total flux quantified by IVIS; ** p = 0.0079 by two-tailed Mann–Whitney test (geometric mean ± geometric SD) (**G**) Percent inhibition of liver infection relative to infection of pVAX control infected mice (geometric mean ± geometric SD). (**H**) Percent of blood stage parasite free mice, determined through blood smears (n = 5 mice/group). **p < 0.01 versus pVAX by Log-rank (Mantel–Cox) test). The positive and negative controls were historical as we have published in Ref.^47^.

We designed DMAb constructs based on CIS43, an anti-CSP human mAb primarily targeting the junctional epitope between the N terminus and the NANP major repeats (Fig. 1A)^21^. A dual plasmid approach where heavy and light chains are encoded onto separate plasmids was used to further improve in vivo expression as we have recently described^33,34^. Additionally, we have observed that reverting specific, non-essential residues in the framework region away from the hypervariable regions back to their germline configuration improves in vivo DMAb expression^33,34,38^. Sequences were analyzed using IMGT DomainGapAlign tool to identify the immunoglobulin gene family as well as sites for potential expression optimization^44–46^. In addition to amino acid changes, codon optimization for expression in murine and human systems was performed, by changing the identity of various nucleotides compared to heavy and light chain variable regions of WT CIS43.

Expi293F cells were transfected and supernatants were harvested to ensure CIS43 GL DMAb had binding activity to CSP. After normalizing transfection supernatants, CIS43 GL exhibited potent binding to recombinant CSP by ELISA (Fig. 1B). To explore the in vivo expression profile of CIS43 GL DMAb, BALB/cJ mice were administered 100 µg of CIS43 GL with co-administration of anti-CD4 and anti-CD8 T cell depletion and serum human IgG titers were monitored over time. Transient T cell depletion was utilized to enable prolonged expression of human IgG in vivo as it curtails the development of mouse anti-DMAb antibodies. This transient CD4+ and CD8+ T cell depletion enables human antibody expression in murine models^32,33^. By Day 21, expression peaked at approximately 67 µg mL^−1^ in serum (Fig. 1C, left). Serum expression of CIS43 GL was durable out to Day 120 post administration (Fig. 1C, right). To elucidate the expression profile of CIS43 GL without CD4/CD8 T cell depletion, mice were administered either 100 µg or 200 µg and expression was monitored at Day 7 (Fig. 1D). The 200 µg dose group demonstrated significantly increased expression; this dose was chosen to advance to a mosquito bite challenge model. These data demonstrate that an anti-malarial DMAb can potently express in vivo and bind recombinant CSP.

### CIS43 GL DMAb protects in a mosquito bite challenge model

To test whether CIS43 GL DMAb is protective in vivo, mice were immunized with 200 µg of CIS43 GL and challenged seven days post DMAb administration (Fig. 1E). Sixteen hours prior to challenge, 100 µg of mAb 311 was administered intraperitoneally as a positive assay control group. On the day of challenge, mice were exposed to the bites of 5 *A. stephensi* mosquitoes infected with *PbPfLuc* transgenic parasites. Forty-two hours post challenge, liver burden was assessed through intraperitoneal administration of d-luciferin. The bioluminescence generated by the transgenic parasites was measured for individual mice via total flux by IVIS Spectrum imager^48^. Both the positive control mAb 311 and CIS43 GL groups showed significantly decreased bioluminescence indicating protection, as compared to the negative control (Fig. 1F). From the bioluminescence data, percent inhibition of liver infection can be calculated relative to infection seen in the pVAX negative control mice. To that end, mice administered recombinant mAb 311 and CIS43 GL DMAb demonstrated 88.7% and 84.4% inhibition of liver infection respectively (Fig. 1G). Importantly, the mAb 311 and CIS43 GL protected mice from developing blood stage infection compared to the negative control group (Fig. 1H). Blood smears taken between day 4 and 10 post challenge showed 80% of mice administered mAb 311 and 60% of mice receiving CIS43 GL achieved protection from blood stage parasitemia. Collectively, these data illustrate that a DNA-encoded anti-malarial antibody CIS43 GL protects mice from blood stage infection in vivo, with potency similar to the related biologically delivered mAb.

### Characterization of 317 GL and L9 GL germline-modified DMAbs

**Figure 2:**
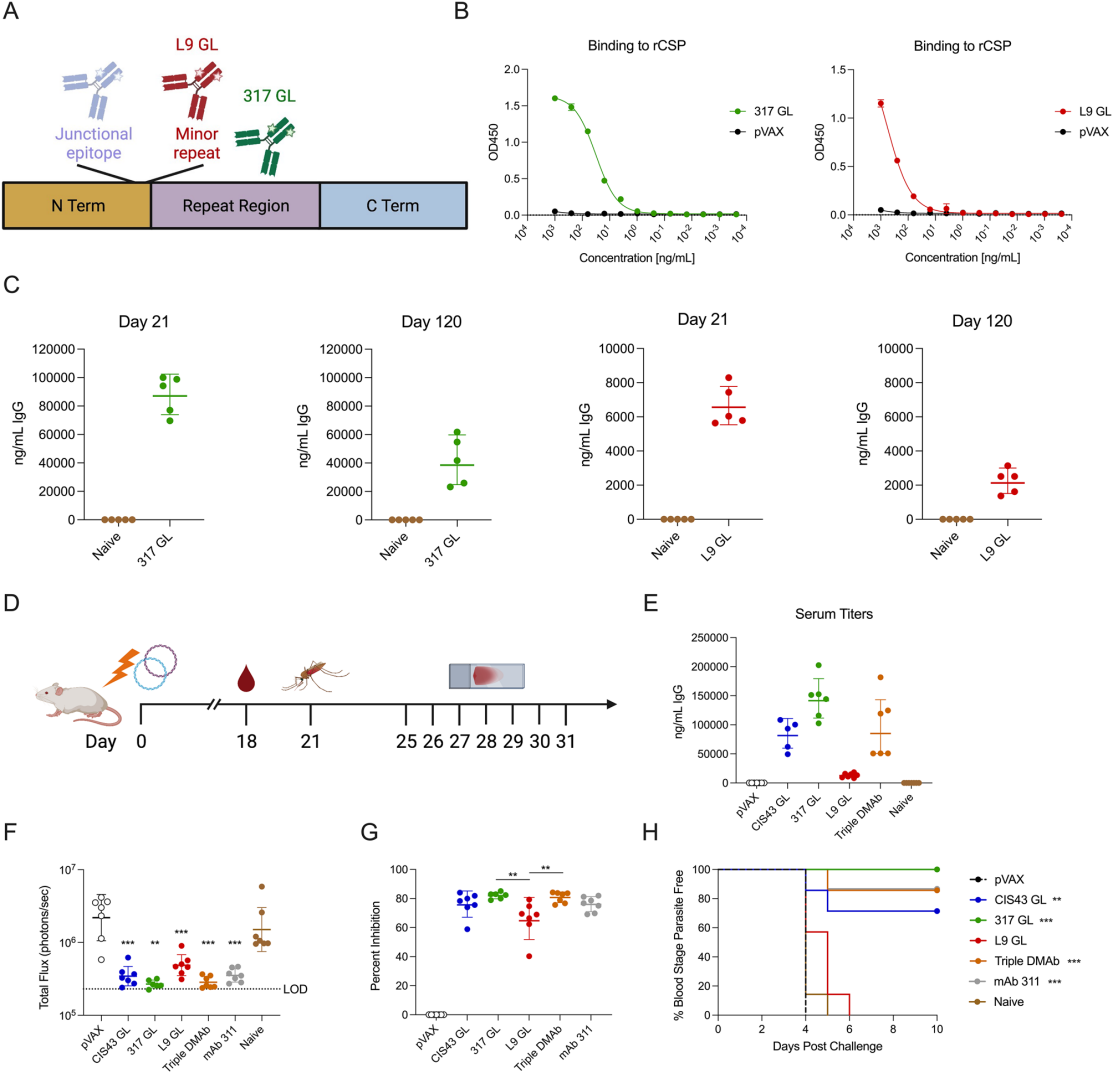
Multiple anti-CSP GL DMAbs protect in mosquito bite challenge. (**A**) Schematic showing 317 GL and L9 GL binding domains on CSP. (**B**) Binding ELISA of pooled supernatants of 317 GL (left) and L9 GL (right) harvested from Expi293F transfection to rCSP (mean ± SD). (**C**) In vivo expression of 317 GL (left two panels) and L9 GL (right two panels) in BALB/c mice at the peak expression timepoint Day 21 through Day 120. Mice were immunized with 100 µg CIS43 intramuscularly in the tibialis anterior with CD4+/CD8+ T cell depletion (n = 5 mice/group; geometric mean ± geometric SD). (**D**) Experimental layout of challenge. Mice were immunized with CIS43 GL (100 µg), 317 GL (100 µg), L9 GL (200 µg) or a cocktail (“Triple DMAb”; CIS43 GL 50 µg, 317 GL 50 µg, L9 GL 100 µg) and challenged 21 days post administration with mosquitos carrying *PbPfLuc* parasites. Positive control recombinant mAb 311 was administered 16 h prior to challenge. Blood smears for blood stage parasitemia were performed from days 4–10 post challenge. (**E**) Serum titers of DMAb 18 days post administration. (**F**) Total flux quantified by IVIS (geometric mean ± geometric SD) (**G**) Percent inhibition of liver infection relative to infection of naive control infected mice (geometric mean ± geometric SD). (**H**) Percent of blood stage parasite free mice, determined through blood smears (n = 6–7 mice/group). Statistical analyses were done using a two-tailed Mann–Whitney test with comparisons relative to naïve infected (**F**,**H**). One-way ANOVA adjusted for multiple comparisons between all treatment groups (**G**). **p < 0.01, ***p < 0.001.

To determine whether other human anti-malarial mAbs with additional specificities could be modified and delivered as DMAbs, we designed germline-modified DMAbs based on human mAbs 317 and L9 (317 GL and L9 GL respectively) targeting the major repeat and minor repeat respectively (Fig. 2A). Variable heavy and light sequences were modified to remove non-essential amino acids in the framework regions. Antibody heavy and light chains were separately encoded into a dual plasmid system. To confirm binding of 317 GL and L9 GL to recombinant CSP, Expi293F cells were transfected and supernatants were harvested for analysis in a binding ELISA. Both 317 GL and L9 GL bound recombinant CSP (Fig. 2B). We studied the expression of the DMAb constructs in vivo using BALB/cJ mice that were administered 100 µg of 317 GL or L9 GL co-administered with anti-CD4/CD8 T cell depletion. Serum expression was monitored using a human IgG quantification ELISA. 317 GL demonstrated strong in vivo expression at approximately 87 µg mL^−1^ at Day 21 with robust titers out to Day 120 (Fig. 2C, left panels). In contrast, L9 GL demonstrated more modest expression at 6 µg mL^−1^ on Day 21, yet titers persisted out to Day 120 (Fig. 2C, right panels). These data demonstrate that multiple anti-CSP human mAbs can be durably expressed in blood after delivery by plasmid DNA.

### Multiple anti-CSP DMAbs protect in a mosquito bite challenge model

To test whether 317 GL and L9 GL DMAbs are protective in vivo, mice were administered 100 µg of CIS43 GL or 317 GL, 200 µg of L9 GL, or a combination of 50 µg CIS43 GL, 50 µg 317 GL, and 100 µg L9 GL. Twice the amount of L9 GL was used relative to CIS43 GL and 317 GL to compensate for lower serum titers generated by this construct, and DMAbs were co-administered with anti-CD4 and anti-CD8 T cell depletion. Mice were challenged 21 days post DMAb administration and blood smears were performed for 10 days starting at Day 25 to monitor for blood stage disease (Fig. 2D). Sixteen hours prior to challenge, 100 µg of mAb 311 was administered intraperitoneally as a positive assay control group. Challenge procedures and readouts using IVIS were done as described above and as reported^48^. Three days prior to challenge, blood was taken to assess serum titers of DMAbs. CIS43 GL, 317 GL, and the triple combination group all showed robust expression in vivo (Fig. 2E). One mouse in the 317 GL group showed very low expression of approximately 1 µg mL^−1^ expression most likely due to a technical issue related to T cell depletion; this mouse was excluded from challenge analyses. Compared to naïve infected mice, CIS43 GL, 317 GL, and the triple DMAb group exhibited similar decreases in total flux compared to the positive control mAb 311 treated animals (Fig. 2F). Similarly, L9 GL also demonstrated decreased total flux although not to the magnitude of the other DMAbs, though its expression levels were considerably lower. All DMAb groups similarly showed robust percent inhibition relative to naïve infected mice, with 317 GL and triple DMAb groups showing a statistically significant increase protection relative to the L9 GL group (Fig. 2G). Blood smears showed 100% of mice administered 317 GL, 87% of mice administered the triple DMAb or positive control, and 71% of CIS43 GL mice were free of blood stage parasitemia 10 days post challenge (Fig. 2H). All mice in the vector control group, naïve infected group, and L9 GL group developed blood stage infection. Collectively, these data demonstrate that multiple anti-malarial DMAbs targeting distinct epitopes are capable of protecting mice from blood stage disease in vivo. Combining DMAbs together however did not show an additional protective effect, likely due to the low serum expression of DMAb L9 GL.

### Characterization of complement knockout variants of anti-malarial DMAbs

**Figure 3:**
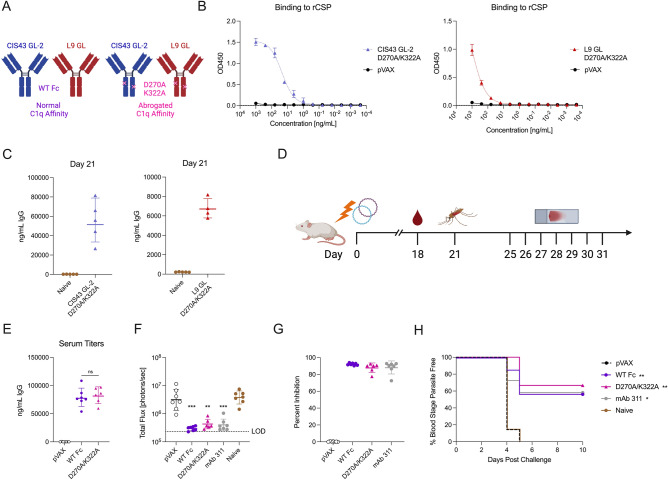
Complement-binding knockout modifications do not impact protection in mosquito bite challenge model. (**A**) Graphical representation of complement modifications of CIS43 GL-2 and L9 GL. (**B**) Binding ELISA of pooled supernatants of CIS43 GL-2 constructs (left) and L9 GL constructs (right) harvested from Expi293F transfection to rCSP (mean ± SD). (**C**) In vivo expression of CIS43 GL-2 D270A/K322A (left) and L9 GL D270A/K322A (right) in BALB/c mice at the peak expression timepoint Day 21. Mice were immunized with 100 µg CIS43 intramuscularly in the tibialis anterior with CD4+/CD8+ T cell depletion (n = 5 mice/group; geometric mean ± geometric SD). (**D**) Experimental layout of challenge. Mice were immunized with CIS43 GL-2 (50 µg) and L9 GL (100 µg) (abbreviated “WT Fc”) or CIS43 GL-2 D270A/K322A (50 µg) and L9 GL D270A/K322A (100 µg) (abbreviated “D270A/K322A”)and challenged 21 days post administration with mosquitos carrying *PbPfLuc* parasites. (**E**) Serum titers of DMAb 18 days post administration. (**F**) Total flux quantified by IVIS (geometric mean ± geometric SD) (**G**) Percent inhibition of liver infection relative to infection of naive control infected mice (geometric mean ± geometric SD). (**H**) Percent of blood stage parasite free mice, determined through blood smears (n = 6–7 mice/group). Statistical analyses were done using a two-tailed Mann–Whitney test between treatment groups (**E**), with comparisons relative to naïve infected (**F**,**H**) or one-way ANOVA adjusted for multiple comparisons between all treatment groups (**G**). **p < 0.01, ***p < 0.001.

We sought to utilize DMAb platform as a tool to test the effect of complement engagement on protective outcomes. As such, we incorporated Fc modifications that ablate IgG hexamerization and subsequent complement deposition. Constructs were developed containing two point mutations in the human IgG1 constant domain: an alanine substitution at position 270 (D270A) and an alanine substitution at position 322 (K322A) (Fig. 3A). These mutations have been used to ablate C1q binding and subsequent complement deposition previously^39,49–55^. Constructs were synthesized, expressed in Expi293F cells, and supernatants were harvested for downstream analyses. Due to the abrogated expression of the D270A/K322A variant of CIS43 GL (Supplementary Fig. 1A), further amino acid modifications were made to the light chain framework region to restore expression (CIS43 GL-2). Constructs baring these new light chains were used in both the D270A/K322A knockout (A/A KO) constructs as well as WT controls. Supernatants containing CIS43 GL-2 A/A and L9 GL A/A were harvested and assessed for anti-CSP binding activity using a binding ELISA. Both CIS43 GL-2 and L9 GL A/A bound recombinant CSP (Fig. 3B, Supplementary Fig. 1B). To determine whether A/A KO DMAbs expressed in vivo, mice were administered 100 µg of either CIS43 GL-2 A/A or L9 GL A/A with CD4/CD8 T cell depleted to observed prolonged DMAb expression. CIS43 GL-2 A/A showed robust expression in vivo at Day 21, while L9 GL A/A showed a more modest expression profile (Fig. 3C). From these data, we conclude that complement knockout Fc modifications of CIS43 GL-2 and L9 GL do not impair binding to recombinant CSP or in vivo expression.

### Complement knockout Fc variants do not impact protection compared to wild-type in a mosquito bite challenge model

To investigate whether the complement knockout Fc variants enhanced protection in a challenge setting, mice were administered 150 µg of total DMAb (100 µg of CIS43 GL-2 and 50 µg of L9 GL) both with either WT Fc domains (“WT Fc”) or knockout Fc domains “D270A/K322A”). Mice were co-administered anti-CD4/CD8 T cell depletion for prolonged DMAb expression. After 21 days, mice were challenged with mosquito bites, liver burden was measured via IVIS, and blood smears were taken 4–10 days post challenge (Fig. 3D). On Day 18, serum was taken to assess in vivo DMAb expression prior to challenge. Both the WT Fc and D270A/K322A DMAb cocktail had similar expression profiles at Day 18 (Fig. 3E). One mouse in the D270A/K322A group died for irrelevant reasons during challenge and was excluded from assessment. As measured by IVIS, total bioluminescence was significantly decreased in the livers of mice receiving the WT Fc cocktail, complement knockout cocktail, or the positive control mAb 311 relative to naïve infected animals (Fig. 3F). As such, percent inhibition relative to naive infected animals was similar in all treatment groups (Fig. 3G). In terms of protection from blood stage parasitemia, both the WT Fc and D270A/K322A KO cocktails conferred comparable protection, with 4 of 7 mice in the WT Fc group and 4 of 6 mice in the D270A/K322A group achieving protection (Fig. 3H). These data demonstrate that Fc variants to ablate complement deposition do not impact protection from blood stage infection in a challenge model with two anti-malarial DMAbs. This indicates that epitope binding is the major mechanism of protection from challenge in this model system.

## Discussion

It is an important goal to develop improved vaccines and prophylactics for malaria. Vaccine efficacy studies have established that the surface protein CSP is a viable sporozoite stage target^56^. The licensed malaria vaccine RTS,S/AS01 targets CSP. However, this vaccine provides a limited protection as over a median four year follow-up period the estimate of protective efficacy in children aged 5–17 months is 28% after three immunizations and 36% after a booster dose provided 20 months after the first immunization. In infants aged 6–12 weeks the efficacy was 18% after three immunizations and 26% after the booster dose^4,9–11^. Trials utilizing CSP-based vaccines have established a possible relationship between their efficacy and anti-CSP antibodies. A distinct correlate of protection is still unknown; these studies focus attention on protection generated by serological immunity to CSP^57–59^. In addition to vaccine candidates targeting CSP, monoclonal antibodies have been isolated from malaria survivors and vaccinees against various CSP epitopes. The central NANP repeat region of CSP is conserved and immunodominant, representing a common target for anti-CSP antibodies^22,60,61^. However, other regions of CSP, such as the junctional epitope bridging the N terminus and NANP repeats, have elicited potent IgG antibodies. Antibodies isolated from vaccinees targeting this junctional epitope demonstrate protection in murine models of malaria^21,62^. Additionally, it has been reported that the junctional epitope, minor repeat, and major repeat are each independently protective and are required for mAbs CIS43, L9, and 317 respectively^63^.

Monoclonal antibodies have been evaluated for infectious disease prophylaxis or therapy. Palivizumab is an FDA-approved humanized murine mAb targeting the F protein of respiratory syncytial virus^64,65^. Additionally, a modified version of CSP junctional-epitope targeting mAb CIS43, CIS43LS, has become the first anti-malarial mAb to complete a Phase I clinical trial. CIS43LS contains mutations designed to increase plasma half-life and has demonstrated efficacy in CHMI models^25^.

Here, we demonstrated that human malarial mAbs administered using a synthetic DNA platform can elicit expression both in vitro and in vivo. We observed that amino acid modifications, as well as encoding heavy and light chains on dual plasmids, allowed for enhanced DMAb expression, showing the importance of in silico modifications for high titer systemic human IgG expression of gene-encoded antibodies. The protective capacity of anti-malarial GL DMAbs have been determined using a well-characterized and rigorous mosquito bite challenge model^22,66,67^. As a control, we compared all DMAbs to recombinant human monoclonal antibody mAb 311; as it has been shown that 311 confers dose-dependent protection in rigorous mosquito bite challenge models^67^. CIS43 GL, 317 GL, and the triple DMAb cocktail were administered 21 days prior to challenge and elicited sterile protection similar to or better than recombinant mAb 311 administered 16 h before challenge. Recombinant mAbs, which are produced in vitro using human cells, are being constantly degraded in vivo post infusion. However, DNA-encoded mAbs are being assembled and secreted from transfected cells in vivo, enabling a unique production and degradation rate. Plasmid DNA delivery via electroporation has demonstrated safety and tolerability in tens of thousands of people in various prophylactic and therapeutic clinical trials^68–71^, and a DNA vaccine for SARS-CoV-2 has been approved under Emergency Use Authorization^72^. DMAbs are a new approach utilizing plasmid DNA as a delivery platform. Additionally, DMAbs leverage the stability, mobility, and safety profile of plasmid DNA, allowing for potential distribution in cold-chain limited settings as compared with recombinant IgG^73^. In vivo expression of DMAbs has been reported in rodent, pig, and NHP models^35,74^. Based on these preclinical data, future studies to address safety and potency of the DMAb platform in humans may be of importance.

In this study, complement abrogating Fc mutations on anti-CSP human DMAbs did not associate with increased or decreased protection. We sought to leverage the DMAb platform as a specific tool to test the effect of complement on sporozoite stage immunity mediated by monoclonal antibodies. Mutations to enhance or abrogate complement activity may be especially useful in the context of merozoite antigens. Multiple antigens, particularly in the merozoite stage of the parasitic life cycle, are particularly susceptible to complement^75^. It has been demonstrated that human mAbs that are able to fix complement to merozoites correlate with protection^76^. The data presented here support that substantial protection can be mediated by direct CSP relevant epitope binding in challenge models. There is still a possibility for the role of complement-independent FcR mediated opsonization. However, early studies have shown that Fab monomers of anti-CSP antibodies are also protective in vivo as with the entire IgG^77^.

Previous studies have demonstrated that DMAb cocktails can be delivered to target multiple different antigens on the same pathogen^33,36^. Such an approach could be employed to target multiple antigens in the same or different stages in the parasitic life cycle. While monoclonal antibody infusions may provide short term protection for an activity such as travel, an anti-PfCSP DMAb such as CIS43 GL or 317 GL could be useful due to its potential for long-term transient expression. In addition, a combination vaccine and immunotherapy approach using a vaccine with a DMAb could provide short- and long-term field immunity, as initial studies have demonstrated with a DNA vaccine and DMAb targeting chikungunya virus^37^.

This study demonstrates the potential of synthetic DNA delivered mAbs targeting PfCSP for malaria prophylaxis. This is the first demonstration of a DNA-encoded antibody as malaria prophylaxis, an approach which potentially provides benefits such as prolonged serum half-life as well as protection in a transient fashion. Leveraging the safety profile of DNA vaccines in humans as well as recent advances in DNA vaccine technology, the DMAb platform allows for rapid design, in silico modifications, and in vivo production of antibody. Systemic expression of human IgG if established to be safe and potent in humans could represent a valuable tool for malaria elimination.

## Methods

### DMAb construction and plasmid synthesis

The sequences used for all DMAbs were derived from the sequences for anti-PfCSP monoclonal antibodies CIS43 (PDB 6B5M), 317 (PDB 6AXL), and L9 (GenBank MT811865 and MT811893). The nucleotide sequences for both heavy and light chain Fab and Fc regions of each clone were codon-optimized for mouse and human to enhance transgene expression. In addition, N terminal framework modifications were introduced in both heavy and light chains. Based on an analysis using the IGMT DomainGapAlign Tool, select amino acid residues in the framework region were reverted back to the germline immunoglobulin gene sequence of highest similarity^44–46^. The optimized HC and LC sequences were inserted into a single plasmid or dual plasmid system with HC and LC encoded on separate plasmids. In either case, a pVAX plasmid DNA expression vector was used. The genes were under the control of a human cytomegalovirus promoter as well as a bovine growth hormone polyA. The HC and LC were encoded separately into individual pVAX expression vectors.

### Cell lines and transfection

Expi293F transfection kit (Thermo Fisher Scientific, Waltham, MA, USA) was used for all transfections. Protocol was followed per manufacturers specifications. Briefly, Expi293F cells were maintained in Expi293 Expression Medium. Cells were incubated on an orbital shaker at 37 °C in 8% CO_2_ conditions. For transfection, 2 × 10^6^ Expi293F cells were plated in 2 mL Expression Medium per well above 95% viability. DNA plasmids were added to Opti-MEM media separately from ExpiFectamine transfection reagent. After a 5 min incubation, the DNA and ExpiFectamine were combined for 20 min to allow for complexation. The DNA plasmid complex was then added to Expi293F cells. After 18 h, Transfection Enhancers were added. Three days after the addition of Transfection Enhancers, cell supernatants were collected for further experimentation.

### Quantification ELISA

For quantification of transfection supernatants and sera, ThermoFisher MaxiSorp 96-well plates (Thermo Fisher Scientific, Waltham, MA, USA) were coated with 5 µg mL^−1^ goat anti-human IgG-Fc (Bethyl Laboratories, Montgomery, TX, USA) overnight at 4 °C. The following day, each plate was washed with PBS (Corning Inc., Corning, NY, USA) + 0.01% Tween-20 (ThermoFisher, Waltham, MA, USA) (PBS-T) four times (4×). Plates were then blocked with 5% milk in PBS for 2 h at RT. Upon completion of blocking, plates were washed again 4× with PBS-T and samples diluted in 1% NCS in PBS-T were transferred to plates for a 1 h incubation at RT. A standard curve was generated using purified human IgGκ or IgGλ (Bethyl Laboratories, Montgomery, TX, USA). Plates were subsequently washed and goat anti-human IgGκ or IgGλ HRP-conjugated secondary antibody (Bethyl Laboratories) was diluted to 1:10,000 and transferred onto plates for 1 h at RT. After secondary incubation, plates were washed 4× and developed using OPD Substrate Tablets (Thermo Scientific) for 10 min in the dark and stopped with 2 N H_2_SO_4_. The Biotek Synergy 2 plate reader was used to read absorbance at 450 nm.

### Binding ELISA

For binding detection of transfection supernatants and sera, the ELISA protocol is as described above. However, recombinant CSP (rCSP) at a concentration of 1 µg mL^−1^ was used as coat protein. The secondary antibody used was HRP-conjugated goat anti-human Fc (Bethyl Laboratories). In addition, 1-Step™ Ultra TMB (Thermo Scientific) was used as detection substrate and allowed to incubate for 5 min before quenching with 2 N H_2_SO_4_. Finally, the sera incubation occurred for 1 h at 37 °C rather than at RT. The Biotek Synergy 2 plate reader was used to read absorbance at 450 nm.

### Animal experiments and immunizations

Female BALB/cJ mice at 6–8 weeks of age were purchased from the Jackson Laboratory. Animal experiments were conducted under protocol #201236 where activities and ethics were approved by the Wistar Institute Institutional Animal Care and Use Committee (IACUC). Challenge studies were performed at Johns Hopkins University (JHU) under IACUC number MO16H35 where activities and ethics were approved by JHU. All methods were performed in accordance with the relevant guidelines and regulations set forth by the Guide for the Care and Use of Laboratory Animals and the Wistar/JHU IACUC committees. Immunizations and challenge are reported according to ARRIVE guidelines. All animals were housed in the Wistar Institute or JHU Animal Facilities.

For the in vivo expression experiments, mice were immunized with DNA plasmids intramuscularly (IM) in the left and right tibialis anterior (TA) muscle and/or left and right quadriceps. A maximum of 50 µg of DNA was administered at any particular site in the muscle. A total of 100 µg or 200 µg of DMAb plasmid was administered depending on the study, with a maximum DNA dose of 100 µg per leg split between the tibialis anterior and quadriceps. DNA mixed in sterile water was co-formulated with hyaluronidase (200 U/L, Sigma Aldrich, Saint Louis, MO, USA) at a 1:1 ratio. A total of 30 µL was injected IM at each site. After IM injection, mice were electroporated at each injection site using the CELLECTRA 3P adaptive electroporation device (Inovio Pharmaceuticals, Bluebell, PA, USA). For studies with CD4+ and CD8+ T cell depletion, 200 µg each of anti-CD4 (Bio X Cell clone GK1.5) and anti-CD8 (Bio X Cell clone YTS169.4) mAbs were administered intraperitoneally 5 min prior to DMAb administration to avoid an anti-human antibody response as we have described^32,33^. Exclusion criteria apply only to challenge if a mouse did not demonstrate detectable DMAb expression as stated above. Mice were not randomized and investigators were not blinded.

### Mosquito bite challenge model

Sixteen hours prior to challenge, the positive control group received 100 µg of mAb 311 intraperitoneally. On the day of challenge, mice were challenged as described^48^ by exposure to the bites of 5 *Anopheles stephensi* mosquitos infected with transgenic *Plasmodium berghei* sporozoites expressing full-length *Plasmodium falciparum* CSP, as well as a luciferase reporter to visually quantify liver parasite burden (*PbPfLuc*). Following challenge, mosquitos were evaluated for being positive for blood meal. Liver parasite burden was examined 42 h post mosquito bite challenge using IVIS Spectrum In Vivo Imaging System (PerkinElmer). Mice were intraperitoneally administered 100 µL of d-Luciferin at a concentration of 30 mg mL^−1^ prior to isoflurane anesthetization and imaging using IVIS Spectrum. Readout was bioluminescence expressed by the *PbPfLuc* parasites as measured in photons/s. Beginning at four and proceeding daily until ten days post challenge, blood smears were conducted to determine the appearance of blood-stage infection. For the challenge in Fig. 1, the positive and negative controls were historical as we have published in Ref.^47^.

### Statistical analyses

All statistical computations were performed using PRISM v8.4.3. To achieve statistical significance in animal expression and challenge studies, n = 5 mice/group was used. Kaplan–Meier survival curves were used to present percent blood-stage parasitemia-free mice in challenged groups. We performed ordinary one-way analysis of variance (ANOVA) to determine statistical significance between groups of three or more (Tukey’s multiple comparison test) where necessary, as well as two-tailed Mann Whitney Tests where treatment groups were compared to negative control. Percent inhibition of liver infection was calculated by dividing the geometric mean bioluminescence of a treatment group by the geometric mean bioluminescence of the pVAX negative control and subtracting the result from 1. Survival curves were analyzed using Log-rank (Mantel Cox) test. For these data to be considered statistically significant, p < 0.05.

## Supplementary Information

Supplementary Figures.

## Data Availability

Data will be made available upon request to the corresponding author though may be subject to a Material Transfer Agreement between institutions.
