# Supplementary material for: Engineered DNA-encoded monoclonal antibodies targeting Plasmodium falciparum circumsporozoite protein confer single dose protection in a murine malaria challenge model
Source: Sci Rep. 2022 Aug 22;12:14313. doi: 10.1038/s41598-022-18375-6 (PMC9395511; doi:10.1038/s41598-022-18375-6)
Supplement: Supplementary file 1 — Supplementary Figures. [file 41598_2022_18375_MOESM1_ESM.pdf]

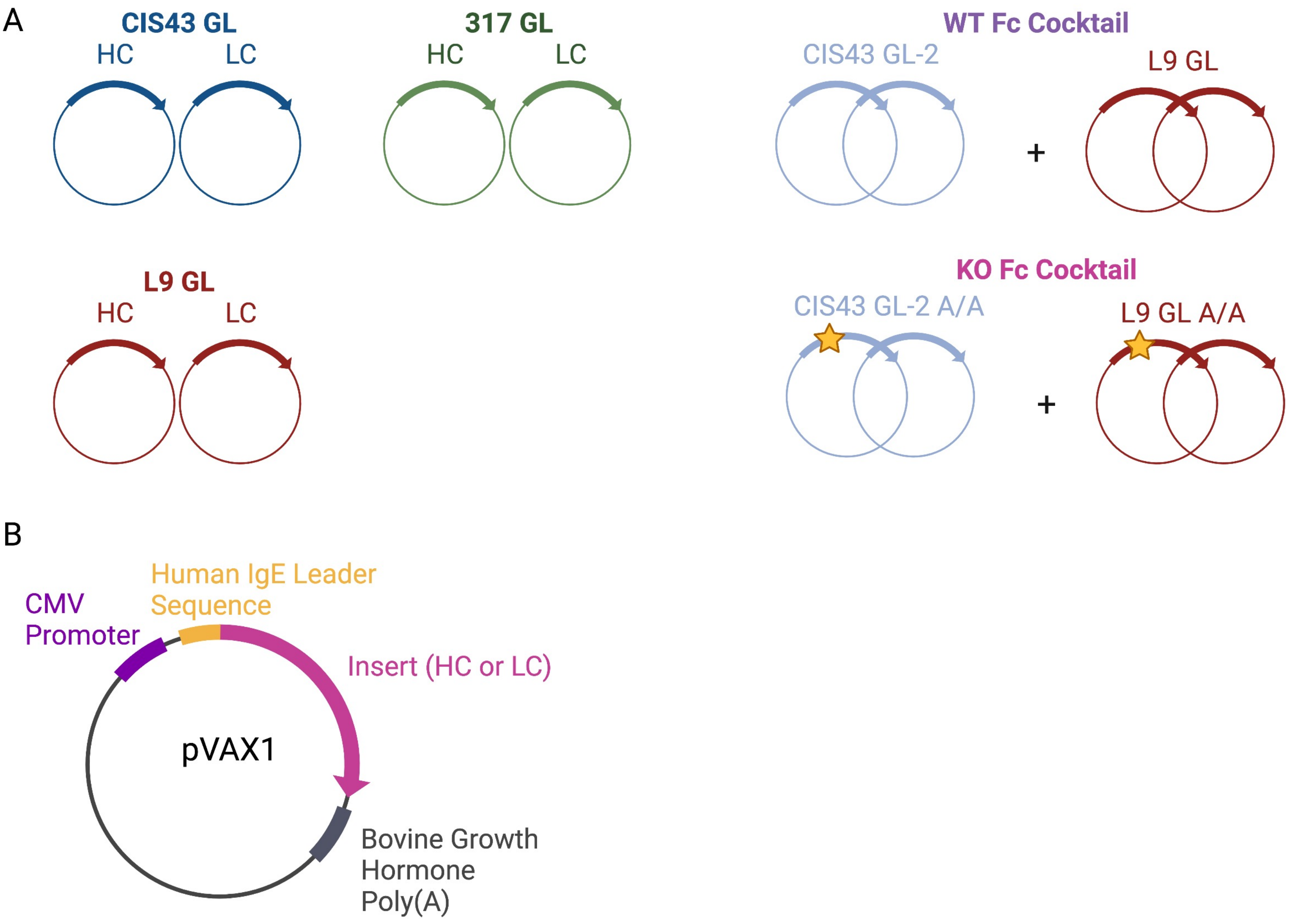

Supplementary Figure 1. ***Vector maps of DMAb constructs***. (A) Schematic of each of the DMAb constructs and DMAb cocktails. (B) Vector map of sample gene in pVAX1.

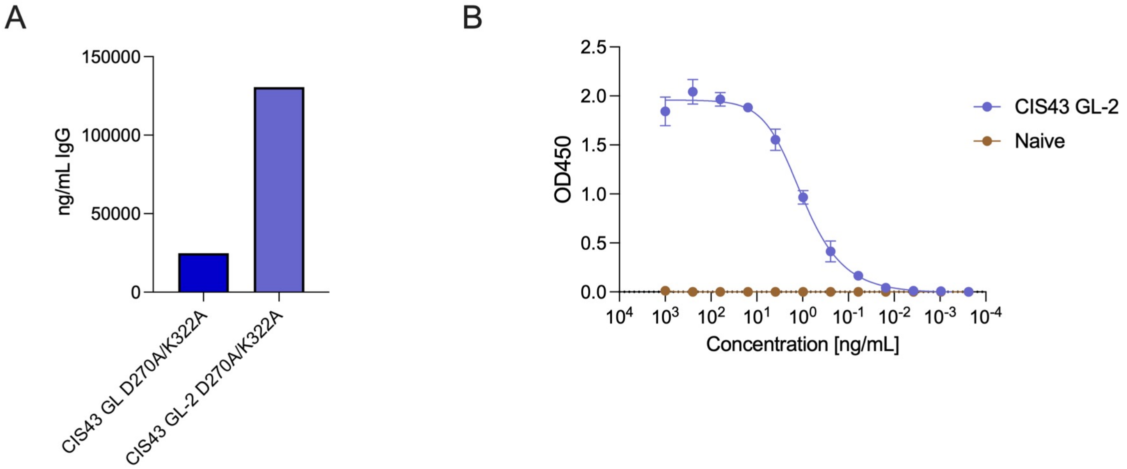

Supplementary Figure 2. ***Characterization of second germline-modified variant of CIS43*****.** (A) Quantification ELISA of pooled serum from transfection supernatants comparing CIS43 GL and CIS43 GL-2 each with D270A/K322A complement abrogating mutations. (B) Binding ELISA to rCSP of pooled serum from mice immunized with CIS43 GL-2 (mean $\pm$ SD)

Confidential
